# Nursing home staffing and operations in Taiwan and the United States: A comparative pilot study

**DOI:** 10.1016/j.ijnsa.2026.100514

**Published:** 2026-02-22

**Authors:** Ya-Wen Lee, John Harris, Ruth A. Anderson, Bianca Shieu

**Affiliations:** a Graduate Institute of Clinical Nursing, College of Medicine, National Chung Hsing University, Taichung 40227, Taiwan; b Healthcare System Operations Center, Changhua Christian Hospital, Changhua County, 500, Taiwan; c Magee-Womens Research Institute, Pittsburgh, PA 15213, USA; d School of Medicine, University of Pittsburgh, Pittsburgh, PA 15213, USA; e School of Nursing, Duke University, Durham, NC 27710, USA; f School of Nursing, UT Health San Antonio, San Antonio, TX 78229, USA

**Keywords:** Nursing homes, Cross-cultural studies, Qualitative research methods, Workforce, Nursing

## Abstract

**Introduction:**

**:** The global aging population has precipitated a surge in demand for long-term care, particularly in nursing homes. While the United States operates under a multipayer system, Taiwan utilizes a single-payer National Health Insurance program supplemented by Long-Term Care 10-year plan 3.0. Despite these systemic differences, both nations face critical workforce challenges. This study explores how these distinct frameworks influence nursing home operations, specifically comparing workforce characteristics, staffing practices, and medication safety protocols.

**Design:**

**:** This was a comparative, descriptive, qualitative pilot study.

**Methods:**

**:** Data were collected between December 2022 and May 2023 at two hospital-affiliated nursing homes in the Northeastern United States and three Christian hospital-affiliated nursing homes in Central Taiwan. Data included direct observations and semi-structured interviews with 34 nursing staff (12 U.S., 22 Taiwan). Thematic analysis and descriptive statistics were used.

**Results:**

**:** Both participating Nursing homes experienced significant staffing shortages, although ratios differed (U.S. sites: 1:20–30; Taiwan sites: 1:30–40). Resident profiles varied: U.S. sites managed diverse populations, while Taiwan sites served homogeneous residents. U.S. sites employed electronic medication systems with clear error reporting, whereas Taiwan sites relied on inefficient manual, paper-based protocols. Staff training was systematic at the U.S. sites but limited at the Taiwan sites. Notably, COVID-19 attrition patterns diverged: at U.S. sites, turnover was immediate due to burnout, whereas at Taiwan sites, resignations were delayed and post-pandemic.

**Conclusions:**

**:** Systemic and cultural contexts profoundly influence NH operations. While U.S.A. sites benefit from technology and standardized training, Taiwan sites require technological adoption to improve medication safety and workflow efficiency. These preliminary findings highlight critical targets for improvement and establish a foundation for future, larger-scale research to enhance resident care quality and the work-life balance of nursing staff globally.

**What is already known:**

• Nursing homes in both the United States and Taiwan face persistent workforce shortages that threaten the quality of long-term care and resident safety.

• The United States and Taiwan operate under distinct healthcare frameworks, with the U.S. relying on a complex multipayer system and Taiwan utilizing a single-payer National Health Insurance program integrated with a national Long-Term Care 10-year plan 3.0.

• Previous comparative research between the U.S. and Taiwan is limited and has primarily focused on macro-level ownership structures rather than granular operational details such as staffing composition, training protocols, or medication administration.

Alt-text: Unlabelled box dummy alt text

**What this paper adds:**

• This qualitative pilot study reveals significant operational disparities, notably that the selected U.S. facilities utilized electronic medication systems and systematic training, whereas the selected Taiwanese facilities relied on manual, paper-based medication protocols and lacked standardized staff education programs.

• The findings highlight distinct resident profiles driven by cultural and systemic factors: the selected U.S. facilities managed a more ethnically diverse population with higher rates of obesity, while the selected Taiwanese facilities cared for a homogenous population with higher medical acuity (e.g., tube feeding, tracheostomies).

• The study identifies contrasting workforce attrition patterns in response to COVID-19, with the U.S. staff experiencing immediate pandemic-related turnover, while the Taiwanese staff exhibited delayed attrition that peaked in the post-pandemic period.

Alt-text: Unlabelled box dummy alt text

## Introduction

The global population is rapidly aging, increasing the demand for long-term care services. In the United States, the population aged 65 and over has grown to 55.8 million over the last decade, classifying the country as an aging society (United States Census Bureau, 2024). Nursing homes are pivotal in providing long-term care for older adults and chronically ill individuals (Shieu et al., 2022). However, Nursing homes in many countries, including the US, face persistent challenges, particularly ongoing nursing staff shortages, which can significantly compromise the quality of care and resident safety (Leland et al., 2024). These demanding environments can also negatively impact the emotional and mental well-being of both residents and staff (Mukamel, Saliba, Ladd and Konetzka, 2023; Tamata and Mohammadnezhad, 2023).

The systemic context for addressing these challenges differs significantly across nations. The United States has a complex, multipayer health care system without universal health coverage, and its long-term care financing relies on a mix of Medicaid, private insurance, and out-of-pocket payments. This structure, compounded by limited oversight and enforcement regarding the allocation of government reimbursements, creates an environment where funds may be diverted toward profits rather than maintaining optimal staffing levels and competitive compensation (Grabowski, O'Malley and Barhydt, 2007; Werner and Coe, 2021); others point to significant oversight issues regarding how these reimbursements are allocated. Recent reports suggest that, despite funding complaints, resources may be diverted to hidden profits, often through "tunneling" to related-party companies rather than to patient care (Gandhi and Olenski, 2024; Harrington, Ross and Kang, 2015). Broadly speaking, the U.S. long-term care landscape also includes home care and community services. In contrast, Taiwan implemented a single-payer National Health Insurance program that provides universal health coverage (Ministry of Health and Welfare of Taiwan, 2025a), later supplemented by a national Long-Term Care 10-year plan 3.0, aimed at creating an integrated and accessible long-term care system (Ministry of Health and Welfare of Taiwan, 2025c) . Taiwan's approach, detailed in policy documents like the 2025 White Paper on Health and Welfare Policy (Ministry of Health and Welfare, 2018), encompasses home-based, community-based, and residential long-term care.

This stark contrast between the U.S. and Taiwan in national healthcare and long-term care policy frameworks offers a valuable opportunity to examine how different systemic approaches may influence nursing home operations, particularly workforce characteristics and staffing practices. Comparative research on the nursing home workforce between the U.S. and Taiwan is limited. There is a particular scarcity of data on staffing composition, training levels, and operational procedures, like medication administration, whereas previous studies have focused mostly on ownership structures within each country separately (Rice, Rosenau, Unruh and Barnes, 2020; Wu, Majeed and Kuo, 2010). Understanding differences and similarities in the nursing home workforce is crucial, as staffing is a key determinant of care quality.

This pilot study conducted a preliminary, qualitative comparison of selected hospital-affiliated nursing homes in the Northeastern United States and Central Taiwan. Specifically, the study team sought to explore and compare facility-level information on resident characteristics, medication administration protocols, staff training and roles, and staffing levels and challenges for nurses within these facilities. This comparative analysis will deepen understanding of global challenges in geriatric nursing workforce management and may identify best practices to optimize staffing and medication safety in resource-constrained environments.

## Methods

### Study design, setting, and sampling strategy

This is a comparative, descriptive, qualitative design pilot study. The settings for this study included two hospital-affiliated nursing homes in the Northeastern region of the U.S. and three general Nursing homes affiliated with Christian hospitals in Central Taiwan. These nursing home sites were selected purposively based on the researchers' ability to secure access through existing professional affiliations. The site selection strategy was crucial to the feasibility of this international comparison and enabled an in-depth exploration suitable for a pilot investigation. Furthermore, the researchers' prior experiences in these national and regional healthcare contexts provided valuable background knowledge, aiding the interpretation of preliminary data and the identification of key areas for comparison relevant to nursing home staffing.

### Data source

A team member (BS) conducted qualitative observations and semi-structured interviews at two hospital-affiliated Nursing homes in the U.S. Another member (YL) conducted similar qualitative observations and semi-structured interviews at three general Nursing homes affiliated with Christian hospitals in Central Taiwan. The study team obtained informed consent from all participants prior to data collection.

Both investigators spent approximately 8 h per day in the nursing homes during the data collection period. Observations were guided by the direct observation guidelines for health researchers outlined by Fix et al. (2022) and were documented using field notes composed after each session. Semi-structured interviews were conducted with available nursing home staff (e.g., Registered Nurse or Licensed Practical Nurse). Examples of interview questions included but were not limited to: "What is your experience providing care to elderly patients?", "Describe your experiences in the documentation and medication administration processes?", and "How many days and what shift do you normally work?" Each interview lasted between 30 and 45 min, was audio recorded with participant permission, and was transcribed verbatim. [Interviews were conducted in English (US) and Mandarin (Taiwan)]. Additionally, investigators collaborated with administrators to collect anonymized aggregate data on resident demographics.

All data collection activities occurred between December 2022 and May 2023, following approval from the Institutional Review Boards (IRBs) of the researchers' institutions.

### Participants

Participants were NH staff who met the following criteria: 1) 18 years of age or older, 2) registered nurse or licensed practice nurse, 3) had worked in a nursing home for at least 6 months, and 4) able to speak English or Mandarin.

### Data analysis

Qualitative data from the semi-structured interviews were analyzed using thematic analysis, facilitated by ATLAS.ti (version 9; Scientific Software Development GmbH). Interview transcripts were imported into the software. Two researchers (BS and YL) used an initial codebook to code the data and then collaboratively grouped codes into potential themes related to nursing home staff experiences. Memos were used throughout the coding process to document reflections and emergent ideas. Discrepancies in coding or theme development were resolved through discussion among the reviewers, with input from another researcher (JH) as needed to achieve consensus.

Descriptive data regarding facility characteristics (e.g., ownership structure, bed capacity) and aggregated residents (e.g., ethnicity, admission/acuity) and staff demographics (e.g., racial/gender distribution, professional licenses, years of experience) were summarized using frequencies Table 1.

**Table 1:** Characteristics of the studied nursing homes.

Characteristic	Taiwan					USA	
	NH_1	NH _2		NH_3		NH_1	NH_2
Ownership Style							
	Christian-based Hospital Affiliated NHs					Not-for profit Hospital Affiliated NHs	
**Ethnicity/ of Nurses**							
	Taiwanese	Taiwanese		Taiwanese		Asian (Taiwanese, Korean, Filipino) Black, Hispanic, White	Asian (Taiwanese), Nepalis, Pacific Islander; White
**Ethnicity/Race of Nurse Aides**							
	Taiwanese, Vietnamese	Taiwanese, Vietnamese		Taiwanese, Vietnamese		Asian, Black, White, Hispanic	Black, Nepalis, Pacific Islander, Guatemalan, White
**Gender**							
Male	0		0		0	8	0
Female	9		9		11	51	15
**Types of licenses**							
RN	7		9		11	32*	9
LPN/LVN	2		0		0	27*	6
**Years of Nursing Experiences**							
	RN: Ranges from1 to 17years LPN: 11 and 18 years		RN: Ranges from 2 to 20 years		RN: Ranges from 2.6 to 23.8 years	RN: Ranges from 9 to 15 years LPN: 3 to 13 years	RN: Ranges from 7 to 27 years LPN: 2 to 10 years
**Bed size of the facility**							
	87		85		104	150 to 200	20

Note: * Include travel and agency nurses. NHs=nursing homes; RN=Registered Nurse; LPN/LVN=Licensed Practical Nurse/ Licensed Vocational Nurse.

A comparative analysis was then performed to identify similarities and differences between the selected Nursing homes in Taiwan and the U.S. This comparison integrated findings from both the qualitative themes (e.g., experiences with care provision, documentation, work environment) and the descriptive characteristics outlined above. Key comparative findings were summarized in matrix form (Table 2, Table 3).

**Table 2:** Similarities between studied nursing homes.

Aspect	Similarity
Ownership Style	Both were hospital-affiliated
Resident Age	Majority of residents were aged 65 and above in both
Staffing	Both experienced severe staff shortages
	Nurses in both typically cared for 20–40 residents per shift.

* Based on the provided information, and there may be additional similarities and differences between nursing homes in the US and Taiwan that were not mentioned.

**Table 3:** Differences between studied nursing homes in USA and Taiwan.

Aspect	Difference
Facility Level Information on Resident Ethnicity	Diverse population (USA) vs. Homogeneous population (Taiwan)
Facility Level Information on Resident Admission/Acuity	Admitted for various conditions, many independent (USA) vs. Admitted for specific high-acuity needs (Nasogastric tube/ Tracheostomy care /Foley catheter care), many require total care (Taiwan)
Facility Level Information on Resident Obesity	Relatively common (USA) vs. Less common (Taiwan)
Medication Administration Protocols	Electronic, start-to-end (USA) vs. Manual, paper-based (Taiwan) Clear procedure exists for medical error reporting (USA) vs. Lack of clear criteria/procedure (Taiwan)
Medication Administration Staff	Administered by one nurse (USA) vs. Divided among different nurses (Taiwan)
Systematic Nursing Staff Training	Provided (USA) vs. Limited (Taiwan)
Nurse Aide Training	Receive nurse aide certificates (implies uniformity) (USA) vs. Varying training backgrounds (esp. foreign aides) (Taiwan)
Staffing During and After COVID-19	Some staff left during COVID-19 peak (USA) vs. Few staff left during COVID-19 peak and many left after pandemic (Taiwan)
Work Schedule	8-hour or 12-hour shifts offered (USA) vs. Typically 8-hour shifts (Taiwan)

* Based on the provided information, and there may be additional similarities and differences between nursing homes in the USA and Taiwan that were not mentioned.

## Results

This preliminary pilot study compared selected Nursing homes in the U.S. and Taiwan across several themes. The analysis revealed both key similarities and differences in their workforce structures, resident characteristics, and operational practices (Table 2, Table 3).

### Participant demographic

For the Taiwan sites, a total of 22 nurses participated in this study. For the U.S. sites, a total of 12 nurses participated. Details are provided in Table 4.

**Table 4:** Sample characteristics of nursing home staff nurses.

Variables	Selected US NHs	Selected Taiwan NHs
Age range (years)		
18–24	0	6
25–34	1	12
35–44	3	4
45–54	2	0
55–64	3	0
65–74	2	0
75 and above	1	0
**Gender**		
Male	1	0
Female	11	22
**Race**		
White	4	0
African American	3	0
Asian	5	22
**Type of Nurse**		
RN	11	22
LPN	1	0
**Working experience (years)**		
0–5	0	16
6–10	5	4
11 and above	7	2
**Bed size of the facility**		
1 to 100	1	16
101 to 300	11	6

Note: RN: Registered Nurse; LPN: Licensed Practical Nurse.

### Similarities between studied nursing homes

Despite systemic and cultural variations, several similarities were identified between the nursing homes studied in the U.S. and Taiwan. Regarding ownership structure, all facilities included in this pilot study were hospital-affiliated. The resident populations in both settings were also similar in age distribution, with the majority reported by administrators to be aged 65 and above. Across both countries, data indicated that many nurses held Registered Nurse qualifications, rather than Licensed Practical/Vocational Nurse certifications.

Operationally, a critical shared challenge emerged regarding staffing: both the U.S. and Taiwan Nursing homes reported staffing shortages. According to the Ministry of Health and Welfare (2025), Taiwan’s official standard for NH mandates at least one registered nurse per 15 residents, with continuous 24-hour coverage. However, our field data revealed a substantial deviation between regulation and practice; participants reported “very high nurse-to-resident ratios (e.g., frequently 1:30 or 1:40, sometimes higher, particularly on night shifts).” In the U.S., the Centers for Medicare & Medicaid Services currently enforces no nationwide minimum staff-to-patient ratio. Instead, the Centers for Medicare & Medicaid Services specifies a total standard of 3.48 h of nursing care per resident per day (HPRD), with a minimum of 0.55 h provided by a Registered Nurse (Center for Medicare and Medicaid Service, 2024). Despite these regulations, participants from U.S. sites similarly reported “high nurse-to-resident ratios (e.g., frequently 1:20 or 1:30), particularly during night shifts."

### Key differences between studied nursing home sites

Alongside these commonalities, numerous significant differences were observed across operational and demographic aspects among the facilities studied.

***Facility-level Information on Resident Ethnicity:*** Resident profiles exhibited notable divergence. Demographic data indicated ethnic diversity among residents at the U.S. sites (majority Caucasian), in contrast to the ethnically homogeneous (Taiwanese) population reported in the Taiwanese facilities. Participants from the Taiwan sites reported “the majority of our residents were born and raised in Taiwan,” whereas participants from the U.S. sites reported “we have residents from Europe, Asia, etc.”

***Facility-level Information on Residents Admission/Acuity***: Differences in resident acuity and reasons for admission were also apparent. Participants from the U.S. sites reported that “we care for residents with diverse conditions where many maintained some independence,” while participants from the Taiwan sites reported that “our sites primarily admitted residents based on specific high-acuity needs (NG tube, trach [care], Foley [catheter care]).” Additionally, participants from the Taiwan sites mentioned that “based on the admission criteria and resident characteristics of the nursing home, the facility primarily serves individuals with chronic illnesses or functional disabilities. Residents with infectious diseases, wandering behaviors, or aggressive tendencies are assessed on a case-by-case basis rather than being categorically inclusive.”

***Facility-level Information on Resident Obesity***: Obesity was relatively common among residents observed in these selected US nursing homes. Participants from the U.S. sites reported that “we have to care for residents that are sometimes over 300 lbs., and we have to be careful not to hurt our back when transitioning them.” In contrast, participants from the Taiwan sites reported that “we usually do not have residents that are overly obese (BMI over 40), but if we do, there is no such equipment provided in the facilities.”

***Medication Administration Protocols*:** Although medication administration protocols varied, they shared a common objective: ensuring patient safety. Participants from the U.S. sites reported that “we utilized an electronic process to manage medication administration. We followed the instructions on the screen and completed each task to ensure patient safety.” In contrast, participants from the Taiwan sites stated that “Our facilities employed a manual, paper-based system. It involved weekly printed medication administration records that required manual updates and sometimes coexist[ed] inefficiently with electronic systems where tasks (e.g., preparation, distribution, documentation) were divided among multiple staff members…Using paper and pencil documentation is very tiring, [and] we have spent a lot of time documenting…”

Medication error reporting processes varied; participants from the U.S. sites described “clear, established protocols”, while those in Taiwan sites reported “a lack of clear criteria and unfamiliarity with formal reporting mechanisms.”

***Systematic Nursing Staff Training***: Nursing staff training approaches and subsequent roles presented contrasts. “Systematic training programs (including orientation and ongoing education)” were reported in the U.S. sites. Conversely, participants from the Taiwan sites reported that “the facilities offer limited systematic training, with education training often relying on hospital-provided in-service or e-learning platforms or peer learning rather than comprehensive, mandated programs.” Additionally, staff from Taiwan sites reported “inadequate or non-facility-provided education related to dementia care**,** sometimes necessitating reliance on prior academic knowledge or personal initiative to seek external courses.”

***Nurse Aide Training***: While nurse aides in U.S. sites underwent standardized certification training, the training backgrounds of aides in Taiwan varied, as participants from Taiwan sites reported that “for individuals recruited from Southeast Asian countries, they often did not receive standardized training.” Furthermore, the scope of practice differed according to observations and staff reports. Participants at U.S. sites said, “some nurse aides also obtain specialized certification to perform advanced tasks (e.g., blood draws)” whereas “Taiwanese aides were generally limited to basic care activities (e.g., bathing, feeding, dressing), although they also routinely handled tasks like measuring vital signs and the final administration of medications.”

***Staffing Attrition Patterns During and After COVID-19.*** A distinct contrast in the timing of staff turnover was observed between the two regions. At U.S. sites, attrition was immediate and acute; staff departures peaked at the height of the pandemic. Participants attributed this to immediate burnout, noting, “My colleagues felt burnout, so they left the healthcare system to find another job instead.” In contrast, Taiwan sites exhibited a pattern of delayed attrition. Staff stability was maintained throughout the crisis, but turnover spiked significantly in the post-pandemic period. As some participants described: “almost everyone stayed and worked during the COVID-19 pandemic, but many have left after the pandemic.”

***Nurse Qualifications:*** Analysis of participant data revealed differences in nurse qualifications. In Taiwan, 100 % of the nurse participants (*N* = 22) held bachelor's degrees in nursing, and all nurse participants in the Taiwan sites were female. In the U.S., 92 % of nurse participants (11 of 12) held bachelor's degrees in nursing; 11 participants were female, and 1 was male.

## Discussion

This pilot study employed a descriptive qualitative approach to compare selected nursing homes in the U.S. and Taiwan, examining differences and similarities in resident characteristics, medication administration protocols, and staffing. Our analysis highlighted key differences and similarities across these domains.

### Resident ethnicity, admission/acuity, obesity, and cultural context

Our findings regarding facility-level information on resident demographics reflect broader national trends and cultural contexts. The observation of predominantly Caucasian residents at U.S. sites aligns with national data, although increasing diversity in U.S. nursing homes is recognized (Centers for Medicare and Medicaid Services, 2015), necessitating culturally competent care approaches. In contrast, the homogeneity of the resident population observed in the Taiwanese sites (primarily Taiwanese/Han Chinese) simplifies certain aspects of care delivery but reflects different societal norms. Taiwanese culture often emphasizes "aging in place" and filial piety (xiao), with families preferring home care when feasible (Hsin and Macer, 2006). This contrasts with the U.S., where institutional placement is more common (Gaugler, 2005), and filial piety is generally less central in mainstream culture. These cultural preferences likely influence not only who resides in Nursing homes but also the observed acuity levels, specifically the higher prevalence of residents with complex medical needs (e.g., tube feeding, tracheostomy, and Foley catheter). The need for total assistance at the Taiwanese sites studied suggests Nursing homes may be utilized primarily when home care becomes unmanageable.

The observed prevalence of obesity and related equipment needs in U.S. sites, compared with their rarity in Taiwanese sites, further highlights differences in population health profiles. These disparities are arguably rooted in long-standing structural and educational differences. For instance, Taiwan emphasizes nutritional literacy early in life; the School Health Act mandates that schools employ dietitians to design menus rich in vegetables and greens, fostering healthy eating habits from childhood (Liu et al., 2015). Furthermore, the built environment in Taiwan generally supports active transportation, such as walking and utilizing public transit, whereas the U.S. infrastructure is heavily reliant on automobiles, contributing to more sedentary lifestyles (Bassett et al., 2008; Zeng et al., 2025). However, this landscape is shifting. Taiwan is currently undergoing a nutrition transition influenced by Western dietary patterns, characterized by an increased consumption of calorie-dense and fried foods (Pan et al., 2011; Muga et al., 2016). This shift poses a long-term risk for rising obesity and cardiovascular disease rates. Consequently, while bariatric equipment is currently less critical in Taiwan than in the U.S., nursing home administrators must remain vigilant of these trends. Future facility planning and procurement strategies should account for the likelihood of a heavier resident population to ensure preparedness for changing demographic needs.

### Medication administration protocols

The medication administration protocols differed considerably between the two countries, with potential implications for efficiency and safety. The U.S. sites utilized electronic systems with a single nurse managing the process, reflecting technology adoption in U.S. healthcare. The manual, paper-based, multi-person segmented approach observed in the Taiwan nursing homes sites (e.g., different staff for packing, distributing, administering, documenting) appeared inefficient and consistent with the literature highlighting medication errors as a significant concern in many Nursing homes across the world (Ferrah, Lovell and Ibrahim, 2017), potentially increasing error risk. The lack of formal protocols for reporting medication errors observed in the Taiwanese sites is particularly concerning, as it hinders quality improvement efforts. This finding highlights a critical area for potential intervention and systematic improvement in the Taiwanese facilities.

### Staffing training, roles, and quality of care

Our findings indicated differences in training and scope of practice. While U.S. sites reported structured orientation and ongoing education, the Taiwanese nursing homes studied lack systematic training programs. This is significant because specialized training is crucial for addressing the complex needs of residents, and inadequate training is linked to poorer care quality (Damery, Flanagan, Jones and Jolly, 2021). The varied training backgrounds of foreign care aides in Taiwan versus the standardized certification required for local aides (and U.S. aides) also present challenges for ensuring consistent care standards.

Furthermore, the role differences described by our participants suggest distinct care delivery models. For instance, while participants in the U.S. reported obtaining specialized certification to perform advanced tasks such as blood draws, those in Taiwan consistently described a scope of practice primarily focused on basic care. Consequently, Taiwanese nurses' workloads extend beyond high resident numbers to include extensive documentation demands (often involving inefficient or non-integrated systems), time-consuming medication rounds, and non-clinical duties such as escorting residents to external appointments. This heavy workload persists despite a highly qualified workforce; the finding that 100 % of participating nurses in the Taiwan sites held bachelor's degrees in nursing (*N* = 22) aligns with research associating higher nurse education levels with better patient safety and quality outcomes (Aiken et al., 2003; Estabrooks et al., 2005; Cramer, Foraita and Habermann, 2012). While encouraging advanced education remains beneficial, its impact is mediated by how roles are structured and supported within the facility, including the need for adequate training for conditions such as dementia.

### Staffing shortages and impact of the COVID-19 pandemic

Staffing shortages emerged as a critical shared challenge, exacerbated by the COVID-19 pandemic in both settings, though with different temporal patterns and responses. National data confirms Taiwan faces declining numbers of nurses and aides in Nursing homes (Ministry of Health and Welfare of Taiwan, 2025b), compounded by restrictive policies on hiring foreign registered nurses (Taiwan Executive Yuan Gender Equality Committee., 2023). While Taiwan relies heavily on foreign aides (primarily from Southeast Asia, with visa limitations), the U.S. nursing workforce historically incorporates more foreign-trained nurses alongside domestic staff from diverse backgrounds (Rovito, Kless and Costantini, 2022) . This diversity in the US workforce can enhance cultural competence, but requires equitable opportunities (Gomez and Bernet, 2019; Nazareno et al., 2021).

Taiwan’s response was heavily influenced by a cultural priority on teamwork and collectivism (Triandis, 2001). During the pandemic, the rallying slogan "one island, same fate" instilled a sense of shared destiny, compelling many healthcare professionals to remain on the frontlines out of moral obligation (Liao et al., 2024; Yu, 2024). However, this prolonged mobilization led to cumulative exhaustion; consequently, many professionals postponed their departure until the immediate crisis subsided, resulting in a significant wave of post-pandemic resignations (Liao et al., 2024).

### Strengths and limitations

This pilot study has several strengths, including a qualitative design that provides rich, contextualized data and a direct comparison between nursing homes in two distinct healthcare systems, facilitated by researcher access. However, findings must be interpreted cautiously due to limitations inherent in a pilot study. The small number of facilities (two in the US, three in Taiwan) selected through purposive, access-based sampling limits the generalizability of findings to all Nursing homes in either country. The specific characteristics of the chosen sites (non-profit U.S., Christian-affiliated Taiwan, and all hospital-affiliated) may not represent the full spectrum of nursing homes. Furthermore, the researcher's familiarity with the sites, while facilitating access, could introduce potential bias in observation or interpretation, which we attempted to mitigate through dual review and discussion.

### Implications and future research

Despite its preliminary nature, this study highlights key areas for potential policy and practice interventions. The inefficiencies and potential safety risks associated with manual medication administration protocols at Taiwanese sites suggest a need for technological adoption and process redesign. The identified gaps in systematic training indicate an urgent need for enhanced educational programs across all staff levels. Shared staffing shortages underscore the global challenge of recruiting and retaining nursing home staff (Jester, 2023), suggesting a need for cross-national learning on workforce strategies that not only address compensation but also prioritize professional development and supportive working conditions that empower staff to deliver safe, high-quality care.

Future research should extend these findings using larger, more diverse samples of nursing homes in both countries, including different ownership types and geographic regions. Qualitative research could examine in greater depth the experiences of foreign care aides in Taiwan and agency nurses in the U.S., and explore cultural influences on care preferences and staff retention. Investigating the implementation and impact of Taiwan's Long-Term Care 10-year plan 3.0 policy on nursing home staffing and quality would also be valuable.

## Conclusions

This comparative pilot study offers initial insights into the complex interplay of systemic, operational, and cultural factors shaping nursing home staffing and care in the US and Taiwan. Our analysis reveals meaningful differences in resident characteristics, medication administration protocols, staffing patterns, and the impact of the COVID-19 pandemic. Collectively, these preliminary findings highlight critical targets for improvement and establish a foundation for future, larger-scale research to enhance the quality of care and life for nursing staff globally.

**Funding:** This study was supported by the Changhua Christian Hospital Research Fund, the Magee-Womens Research Institute Postdoctoral Award, and UT San Antonio Faculty Start-Up Fund.

## CRediT authorship contribution statement

**Ya-Wen Lee:** Data curation, Formal analysis, Funding acquisition, Methodology, Project administration, Software, Visualization, Writing – original draft. **John Harris:** Supervision, Validation, Writing – review & editing. **Ruth A. Anderson:** Writing – review & editing. **Bianca Shieu:** Conceptualization, Data curation, Formal analysis, Funding acquisition, Methodology, Project administration, Software, Visualization, Writing – original draft.

## Declaration of competing interest

None to declare.

## References

[bib0001] Aiken L.H., Clarke S.P., Cheung R.B., Sloane D.M., Silber J.H. (2003). Educational levels of hospital nurses and surgical patient mortality. JAMa.

[bib0002] Bassett D.R., Pucher J., Buehler R., Thompson D.L., Crouter S.E (2008). Walking, cycling, and obesity rates in Europe, North America, and Australia. J. Phys. Act. Health.

[bib0003] Center for Medicare and Medicaid Service (2024). Medicare and Medicaid programs: minimum staffing standards for long-term care facilities and medicaid institutional payment transparency reporting final rule (CMS 3442-F). https://www.cms.gov/newsroom/fact-sheets/medicare-and-medicaid-programs-minimum-staffing-standards-long-term-care-facilities-and-medicaid-0#:~:text=Staffing%20in%20LTC%20facilities%20has,in%20meeting%20the%20minimum%20standard.

[bib0004] Centers for Medicare and Medicaid Services (2015). Nursing home data compendium. https://www.cms.gov/Medicare/Provider-Enrollment-and-Certification/CertificationandComplianc/downloads/nursinghomedatacompendium_508-2015.pdf.

[bib0005] Cramer H., Foraita R., Habermann M. (2012). Nursing errors and the consequences. results of a survey of nurses from inpatient care institutions. Pflege.

[bib0006] Damery S., Flanagan S., Jones J., Jolly K. (2021). The effect of providing staff training and enhanced support to care homes on care processes, safety climate and avoidable harms: evaluation of a care home quality improvement programme in England. Int. J. Environ. Res. Public Health.

[bib0007] Estabrooks C.A., Midodzi W.K., Cummings G.G., Ricker K.L., Giovannetti P. (2005). The impact of hospital nursing characteristics on 30-day mortality. Nurs. Res..

[bib0008] Ferrah N., Lovell J.J., Ibrahim J.E. (2017). Systematic review of the prevalence of medication errors resulting in hospitalization and death of nursing home residents. J. Am. Geriatr. Soc..

[bib0009] Gandhi A., Olenski A. (2024). Tunneling and hidden profits in health care. https://ssrn.com/abstract=4762965.

[bib37] Gaugler J.E. (2005). Family involvement in residential long-term care: a synthesis and critical review. Aging Ment. Health.

[bib0010] Gomez L.E., Bernet P. (2019). Diversity improves performance and outcomes. J. Natl. Med. Assoc..

[bib0011] Grabowski D.C., O'Malley A.J., Barhydt N.R. (2007). The costs and potential savings associated with nursing home hospitalizations. Health Aff. (Millwood).

[bib0012] Harrington C., Ross L., Kang T. (2015). Hidden owners, Hidden profits, and poor nursing home care: a case study. Int. J. Health Serv..

[bib0013] Hsin D.H., Macer D. (2006). Comparisons of life images and end-of-life attitudes between the elderly in Taiwan and New Zealand. J. Nurs. Res..

[bib0014] Jester R. (2023). Editorial - global shortage of nurses - Rebecca Jester for May 2023 issue. Int. J. Orthop. Trauma Nurs..

[bib0015] Leland N.E., Prusynski R.A., Shore A.D., Cary M.P., Falvey J., Mroz T., Saliba D. (2024). Skilled nursing facility staffing shortages: sources, strategies, and impacts on staff who stayed. Health Serv. Res..

[bib0016] Liao Z.Y., Sun S.J., Clarissa C., Aviles L., Lin C.P., Kao C.T., Shih Y.H., Lo Y.S., Chen L.A. (2024). Exploring the challenges of Taiwanese nurses in the COVID-19 post-pandemic era. J. Formos. Med. Assoc..

[bib0017] Liu C.H., Chang F.C., Liao L.L., Niu Y.Z., Cheng C.C., Shih S.F., Chang T.C., Chou H.P. (2015). Expanding school-district/university partnerships to advance health promoting schools implementation and efficacy in Taiwan. Health Educ. Res..

[bib0018] Ministry of Health and Welfare (2018). Taiwan's 2025 White Paper on Health and welfare policy and the indigenous peoples chapter. https://www.mohw.gov.tw/cp-26-42978-1.html.

[bib0019] Ministry of Health and Welfare of Taiwan (2025). 2025 Taiwan health and welfare report. https://www.mohw.gov.tw/cp-137-81831-2.html.

[bib0021] Ministry of Health and Welfare of Taiwan (2025). Official statistics. https://dep.mohw.gov.tw/dos/cp-5223-62358-113.html#_4.%E9%95%B7%E6%9C%9F%E7%85%A7%E9%A1%A7.

[bib0022] Ministry of Health and Welfare of Taiwan (2025). Taiwan’s long-term care 10-year plan 3.0. https://1966.gov.tw/LTC/cp-6572-85008-207.html.

[bib0023] Muga M.A., Owili P.O., Hsu C.Y., Rau H.H., Chao J.C. (2016). Association between Dietary Patterns and Cardiovascular Risk Factors among Middle-Aged and Elderly Adults in Taiwan: A Population-Based Study from 2003 to 2012. PLoS One.

[bib0024] Mukamel D.B., Saliba D., Ladd H., Konetzka R.T. (2023). Association of staffing instability with quality of nursing home care. JAMa Netw. Open..

[bib0025] Nazareno J., Yoshioka E., Adia A.C., Restar A., Operario D., Choy C.C. (2021). From imperialism to inpatient care: work differences of Filipino and White registered nurses in the United States and implications for COVID-19 through an intersectional lens. Gend. Work Organ..

[bib0026] Pan W.H., Wu H.J., Yeh C.J., Chuang S.Y., Chang H.Y., Yeh N.H., Hsieh Y.T. (2011). Diet and health trends in Taiwan: comparison of two nutrition and health surveys from 1993-1996 and 2005-2008. Asia Pac J Clin Nutr.

[bib0027] Rice T., Rosenau P., Unruh L.Y., Barnes A.J. (2020). United States: health system review. Health Syst. Transit..

[bib0028] Rovito K., Kless A., Costantini S.D. (2022). Enhancing workforce diversity by supporting the transition of internationally educated nurses. Nurs Manage.

[bib0029] Shieu B.M., Toles M., Hoben M., Schwartz T.A., Beeber A.S., Anderson R.A. (2022). A Cross-Sectional, Correlational Study Comparing Individual Characteristics of Younger and Older Nursing Home Residents using Western Canadian Resident Assessment Instrument-Minimum Data Set (RAI-MDS) 2.0. J Am Med Dir Assoc.

[bib0030] Taiwan Executive Yuan Gender Equality Committee (2023). https://gec.ey.gov.tw/en/.

[bib0031] Tamata A.T., Mohammadnezhad M. (2023). A systematic review study on the factors affecting shortage of nursing workforce in the hospitals. Nurs. Open..

[bib0032] Triandis H.C. (2001). Individualism-collectivism and personality. J. Pers..

[bib0033] Werner R.M., Coe N.B. (2021). Nursing Home Staffing Levels Did Not Change Significantly During COVID-19. Health Aff (Millwood).

[bib0034] Wu T.Y., Majeed A., Kuo K.N. (2010). An overview of the healthcare system in Taiwan. London. J. Prim. Care (Abingdon).

[bib0035] Yu S.-J. (2024). Unfolding affective atmospheres during the COVID-19 pandemic crisis: the constitution of affective governance in Taiwan. Geoforum..

[bib0036] Zeng Y., Rudge G., Yu T., Chen W., Chong K.C., Huang Y., Thomas G.N., Lao X.Q. (2025). Pavements to longevity: the influence of neighborhood walkability on mortality in Taiwan. Environ. Health Perspect..

